# Assessing LLMs on IDSA Practice Guidelines for the Diagnosis and Treatment of Native Vertebral Osteomyelitis: A Comparison Study

**DOI:** 10.3390/jcm14144996

**Published:** 2025-07-15

**Authors:** Filip Milicevic, Maher Ghandour, Moh’d Yazan Khasawneh, Amir R. Ghasemi, Ahmad Al Zuabi, Samir Smajic, Mohamad Agha Mahmoud, Koroush Kabir, Ümit Mert

**Affiliations:** Department of Orthopaedics and Trauma Surgery, Helios University Hospital, University Witten/Herdecke, 42283 Wuppertal, Germany; filip.milicevic@helios-gesundheit.de (F.M.); mghandourmd@gmail.com (M.G.); mohdyazan.khasawneh@helios-gesundheit.de (M.Y.K.); reza20gh20@yahoo.de (A.R.G.); ahmad.alzuabi@hotmail.com (A.A.Z.); dr.med.smajic@gmail.com (S.S.); m.agha.mahmoud@gmail.com (M.A.M.); koroush.kabir@helios-gesundheit.de (K.K.)

**Keywords:** native vertebral osteomyelitis, large language models, ChatGPT, Gemini, clinical decision support, artificial intelligence, IDSA guidelines, spine infection

## Abstract

**Background**: Native vertebral osteomyelitis (NVO) presents diagnostic and therapeutic challenges requiring adherence to complex clinical guidelines. The emergence of large language models (LLMs) offers new avenues for real-time clinical decision support, yet their utility in managing NVO has not been formally assessed. **Methods**: This study evaluated four LLMs—Consensus, Gemini, ChatGPT-4o Mini, and ChatGPT-4o—using 13 standardized questions derived from the 2015 IDSA guidelines. Each model generated 13 responses (n = 52), which were independently assessed by three orthopedic surgeons for accuracy (4-point scale) and comprehensiveness (five-point scale). **Results**: ChatGPT-4o produced the longest responses (428.0 ± 45.4 words), followed by ChatGPT-4o Mini (392.2 ± 97.4), Gemini (358.2 ± 60.5), and Consensus (213.2 ± 68.8). Accuracy ratings showed that ChatGPT-4o and Gemini achieved the highest proportion of “Excellent” responses (54% and 51%, respectively), while Consensus received only 20%. Comprehensiveness scores mirrored this trend, with ChatGPT-4o (3.95 ± 0.79) and Gemini (3.82 ± 0.68) significantly outperforming Consensus (2.87 ± 0.66). Domain-specific analysis revealed that ChatGPT-4o achieved a 100% “Excellent” accuracy rating in therapy-related questions. Statistical analysis confirmed significant inter-model differences (*p* < 0.001). **Conclusions**: Advanced LLMs—especially ChatGPT-4o and Gemini—demonstrated high accuracy and depth in interpreting clinical guidelines for NVO. These findings highlight their potential as effective tools in augmenting evidence-based decision-making and improving consistency in clinical care.

## 1. Introduction

Spine surgeons frequently face complex diagnostic and therapeutic challenges in the management of spinal disorders, and there is an ever-growing need to adhere to evidence-based clinical guidelines. These guidelines are the keystone of contemporary medicine, underpinning standardized, safe, and effective patient care with less unwarranted variation. Among the numerous spinal ailments that offer a challenge to clinicians, native vertebral osteomyelitis (NVO) or spondylodiscitis stands out for the elusiveness of its presentation and diagnostic nuance. High morbidity and mortality, especially in the increasingly large population of immunocompromised and multimorbid patients, are associated with NVO despite it contributing only 3% to 5% of the total cases of osteomyelitis every year [1].

Native vertebral osteomyelitis most typically occurs as a consequence of hematogenous seeding of infection from distant body sites. *Staphylococcus aureus* remains the most common etiologic pathogen, with Escherichia coli and other Gram-negative organisms also frequently implicated [2]. Despite being a serious illness, NVO is typically underdiagnosed according to its non-specific clinical presentation. Patients may manifest non-specifically with chronic or subacute back pain, constitutional symptoms, or low fevers. These manifestations are commonly attributed to more common degenerative spine conditions, thereby triggering misdiagnosis or protracted delays in commencing treatment. The literature reports that the mean lag time between presentation of symptoms and a positive diagnosis can range from two to six months, highlighting the urgent need for improvement in diagnostic methods [3,4,5]. Challenging diagnosis and management by NVO are compounded by its potential severity. In-hospital mortality in the more recent literature varied rather widely—from 2% to 17%—likely due to both the heterogeneity of patient populations and variability in clinical practice. With timely diagnosis and evidence-based therapy thus being critical to improved patient outcomes, the standardized management approaches were recognized as a need [6]. In this regard, the Infectious Diseases Society of America (IDSA) published comprehensive clinical practice guidelines in 2015 on the diagnosis and treatment of NVO in adults. These guidelines are the most current available at present, based on a systematic evidence review and consensus of experts [7].

However, ongoing advances in science and technology proceeded at a rate quicker than intermittent guideline updates, highlighting the need for more active decision-support systems at the clinical level. In line with this, large language models (LLMs) have had transformative effects in the field of artificial intelligence. Since OpenAI’s launch of ChatGPT on 30 November 2022, LLMs have gained immense attention in both public and professional spheres [8]. These models, including OpenAI’s GPT-4, Google’s Gemini, and the evidence-focused Consensus platform, are trained on vast corpora of data comprising the scientific literature, clinical resources, and publicly available text. Having billions of parameters, such models can learn from context, generate coherent text, and even generate compounded information synthesized in patterns like human thought [9]. The deployment of LLMs in healthcare settings introduces the possibility of real-time, adaptive clinical support. The AI tools are capable of handling vast amounts of information, generating responses that adhere to the state of medical science at a moment in time, and delivering clinicians evidence-based practice-guided decision-making assistance. In this context, LLMs can theoretically function as adjuncts to traditional guidelines, bridging the gap between static documents and dynamic frontline healthcare workers’ needs. However, though their promise is substantial, the application of LLMs to high-stakes domains such as the management of infectious diseases must be scrutinized closely. Diagnosing and treating NVO has extremely high stakes, and any clinical guidance must not only align with the best evidence that is available but also reflect the balanced judgment that functions in extremely complicated clinical situations. It is at this crossroads of innovation and safety for patients (where high stakes are present) that the current study positions itself [10]. The objective of this research is to determine the reliability and consistency of clinical recommendations provided by prominent LLMs—ChatGPT, Gemini, and Consensus—against IDSA’s 2015 guidelines for the management and diagnosis of NVO.

## 2. Materials and Methods

### 2.1. Ethical Considerations

All large language models (LLMs) assessed in this study are publicly accessible tools available to users under standard terms of service. The study adhered to all applicable ethical principles related to research involving artificial intelligence applications in healthcare settings.

### 2.2. Study Design

A cross-sectional comparative analysis was conducted between 9 November 2024 and 25 February 2025, at the Department of Trauma Surgery, Orthopedics, and Sports Medicine, Helios University Clinic Wuppertal, University of Witten/Herdecke, Germany. The primary aim was to evaluate the accuracy and comprehensiveness of clinical recommendations generated by four different large language models by the 2015 Infectious Diseases Society of America (IDSA) clinical practice guidelines for the diagnosis and management of native vertebral osteomyelitis (NVO) [7].

**Figure 1:**
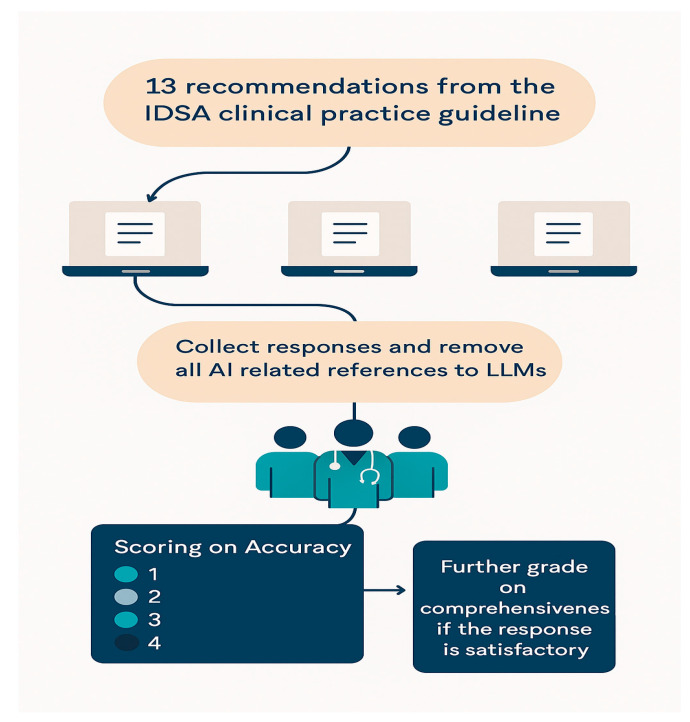
Flowchart of the overall study design.

A total of 13 structured clinical queries were derived directly from the abridged 2015 IDSA guideline for NVO and stratified into three domains, reflecting the key pillars of clinical management: (1) clinical diagnostics, (2) clinical therapy, and (3) clinical follow-up. The full list of clinical questions is provided in Supplementary Table S1. Each question was independently submitted to four LLM platforms: ChatGPT-4o and ChatGPT-4o Mini (OpenAI, San Francisco, CA, USA), Gemini Advanced v3.4.1 (Google LLC, Mountain View, CA, USA), and Consensus Premium v2.7.9 (Consensus AI, Boston, MA, USA). To eliminate potential influence from prior interactions, each question was posed in a fresh, new chat instance. Responses were captured verbatim and stored securely for subsequent analysis. Microsoft Word and Microsoft Excel were employed to tabulate, structure, and prepare data for expert review. A schematic representation of the study workflow is provided in Figure 1.

Each model’s response was independently evaluated by a panel of three board-certified orthopedic surgeons with expertise in spinal infections. These evaluators assessed the responses for consistency with the IDSA guidelines and scored them accordingly.

ChatGPT-4o and ChatGPT-4o Mini (OpenAI) were accessed via the OpenAI web platform under the “Pro” subscription plans during February 2025. Gemini Advanced (Google) refers specifically to “Gemini 1.5 Pro”, accessed through the Google AI web interface in February 2025. Consensus Premium (Consensus AI) was accessed through its web platform (Premium plan) during the same timeframe. Each query was submitted via the respective web application interfaces under default “advanced” or “premium” modes, with no additional plugins or third-party tools.

### 2.3. Accuracy Assessment

The expert grading panel consisted of three senior orthopedic specialists with varying years of clinical experience in diagnosing and managing NVO. Each evaluator was blinded to the identity of the language model to prevent bias. The panel was tasked with independently rating each response using a predefined four-point ordinal scale adapted from prior validated studies:

- (a) Poor: The response contains significant factual inaccuracies that could mislead clinicians and result in potentially harmful clinical decisions.
- (b) Moderate: The response includes moderate factual inaccuracies. While unlikely to result in harm, these responses require clarification to ensure optimal patient care.
- (c) Good: The response contains only minor factual errors and may require limited clarification but is generally safe and clinically usable.
- (d) Excellent: The response is entirely accurate, complete, and aligned with guideline-based recommendations, requiring no clarification.

### 2.4. Comprehensiveness Assessment

In addition to accuracy, the comprehensiveness of LLM-generated responses was also assessed. Only responses rated as “moderate,” “good,” or “excellent” were considered in this secondary evaluation, excluding those deemed “poor” by a majority of the reviewers.

A five-point scale was used to evaluate the depth and detail of each answer:

- (a) Not Comprehensive: The response is grossly deficient and lacks necessary clinical details.
- (b) Slightly Comprehensive: The response offers only minimal and basic information.
- (c) Moderately Comprehensive: The response provides an acceptable level of detail but may omit certain nuances.
- (d) Comprehensive: The response thoroughly covers most of the critical aspects outlined in the guidelines.
- (e) Very Comprehensive: The response demonstrates a high level of detail, incorporating extensive and nuanced information fully in line with IDSA guidance.

These scoring tools were adapted from prior published studies evaluating LLM performance on clinical questions, particularly in spine surgery and diagnostic AI applications [11,12,13,14,15,16,17,18]. The scales were designed through expert consensus among board-certified orthopedic surgeons, ensuring alignment with IDSA guidelines while maintaining clinical applicability. We acknowledge the potential subjectivity inherent in such assessments, which we aimed to minimize through blinded, independent review and consensus scoring.

### 2.5. Statistical Analysis

All statistical analyses were performed using GraphPad Prism software (Version 10.4.1; GraphPad Software, Boston, MA, USA). To assess differences in character count across the four LLM responses, a one-way analysis of variance (ANOVA) was conducted, followed by Tukey’s post hoc test where applicable. Since word count, accuracy scores, and comprehensiveness ratings did not conform to normal distributions, non-parametric tests were employed. For comparing word counts and ordinal evaluation scores (accuracy and comprehensiveness), the Kruskal–Wallis Rank Sum test was used, followed by Dunn’s multiple comparisons post hoc test to identify pairwise differences. Proportional differences in scoring categories (e.g., excellent vs. good vs. moderate) among the four LLMs were analyzed using a two-tailed Pearson’s chi-square (χ^2^) test with Fisher’s exact correction where expected cell counts were low. Multiple hypothesis testing was accounted for using the Bonferroni correction to adjust the *p*-values, ensuring control of the family-wise error rate. Inter-rater agreement among the expert panel was assessed using Fleiss’ Kappa (κ) statistics and per cent agreement. A *p*-value less than 0.05 was considered statistically significant for all comparisons. This multi-pronged methodological approach ensured a robust and reproducible assessment of each LLM’s performance against the established gold-standard clinical guidelines issued by the IDSA.

## 3. Results

We analyzed the responses generated by four LLMs—ChatGPT-4o, ChatGPT-4o Mini, Gemini, and Consensus—in reply to 13 standardized clinical questions related to NVO. Each response was evaluated in terms of length, accuracy, and comprehensiveness. ChatGPT-4o consistently produced the most verbose answers, with a mean word count of 428.0 ± 45.4 and a character count of 2705.0 ± 295.3. In descending order of length, it was followed by ChatGPT-4o Mini (392.2 ± 97.4 words; 2551.0 ± 175.6 characters), Gemini (358.2 ± 60.5 words; 2223.0 ± 106.7 characters), and Consensus (213.2 ± 68.8 words; 1313.0 ± 427.2 characters) (Table 1 and Table 2).

When examining response accuracy, a Kruskal–Wallis test revealed significant differences among the models (χ^2^(3) = 22.76, *p* < 0.001). Gemini achieved the highest average rating (3.49 ± 0.56), closely followed by ChatGPT-4o (3.41 ± 0.72), ChatGPT-4o Mini (3.31 ± 0.57), and Consensus (2.87 ± 0.52). Post hoc comparisons confirmed that Consensus scored significantly lower than all other models (*p* < 0.05 for all), while no significant differences were observed between Gemini, ChatGPT-4o, and ChatGPT-4o Mini.

**Figure 2:**
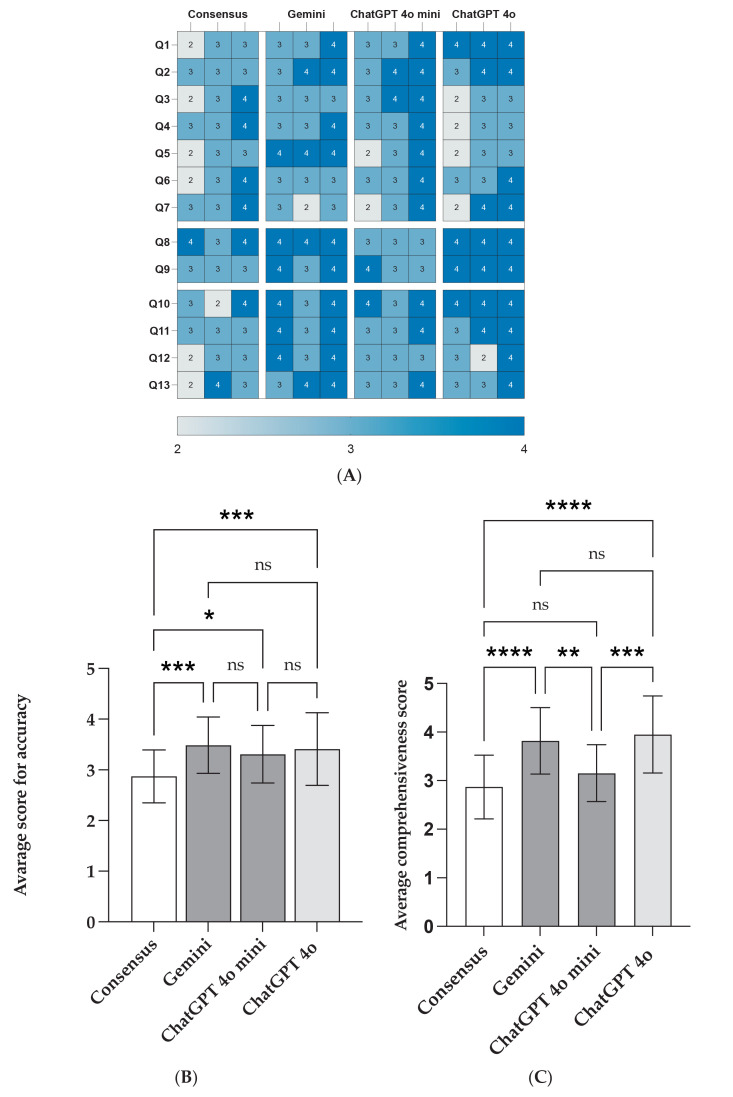
Average accuracy scores of LLM-chatbot replies to ISDA-related queries, as determined by the three orthopaedic graders. Heatmap (**A**) of individual accuracy score, (**B**) average scores for accuracy, and (**C**) average comprehensiveness scores. The asterisks indicating statistical significance.

**Figure 3:**
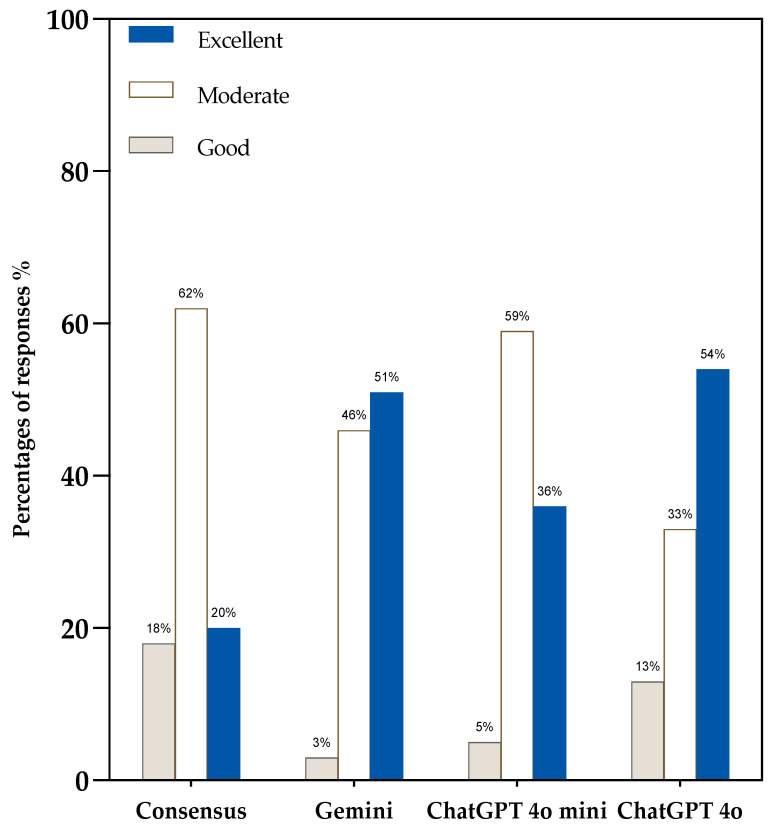
Consensus-based accuracy ratings of LLM-chatbot responses to IDSA practice guidelines related questions. LLM = Large language model. IDSA = Infectious Diseases Society of America.

Visual representations further clarified these findings. Figure 2 includes a heatmap of individual grader responses (A), a summary of average accuracy ratings (B), and a comparison of comprehensiveness scores (C). Additionally, the consensus-based accuracy ratings in Figure 3 reveal that ChatGPT-4o and Gemini achieved the highest proportions of “Excellent” responses (54% and 51%, respectively). In contrast, ChatGPT-4o Mini and Consensus received fewer “Excellent” ratings, with 36% and 20%, respectively. Notably, ChatGPT-4o had the lowest combined share of “Good” or “Moderate” scores, reinforcing its reliability.

A domain-level breakdown of accuracy revealed further distinctions. ChatGPT-4o performed exceptionally in clinical therapy, receiving an “Excellent” rating in 100% of responses in this category. Gemini also showed robust performance in therapy (78% Excellent) and follow-up (67% Excellent). ChatGPT-4o Mini was most consistent in follow-up (78% Good), whereas Consensus responses were distributed more conservatively across all domains Table 3.

The comprehensiveness of the LLM-generated answers was also analyzed. ChatGPT-4o and Gemini received the highest average scores (3.95 ± 0.79 and 3.82 ± 0.68, respectively), significantly outperforming ChatGPT-4o Mini (3.15 ± 0.57) and Consensus (2.87 ± 0.66). These differences were statistically significant (*p* < 0.001), indicating that higher-performing models not only produced more accurate responses but also provided richer clinical detail. No significant differences were observed between the top-tier models, nor between the lower-performing ones (Table 4).

Inter-reviewer agreement for accuracy and comprehensiveness ratings is shown in Table 5. Fleiss’ Kappa indicated moderate-to-substantial agreement among reviewers.

## 4. Discussion

The release of ChatGPT by OpenAI in November 2022 marked the break-even point for the application of LLMs, generating widespread interest from both the academic and general communities. The innovation has since stimulated the development of numerous sophisticated LLMs for dedicated purposes. As interest in their incorporation into scientific and clinical environments grows, the impact of the tools in healthcare workflows remains a current area of investigation [19,20].

As these models mature, it is increasingly important to validate their performance with accuracy in terms of completeness, safety, and accuracy in clinically applicable environments. Though LLMs are capable of generating vast quantities of text, their reliability for use on detailed medical guidelines, particularly when applied to rare conditions like NVO, must be rigorously investigated. Clinical release without vetting has the potential to inject misinformation, idealistic advice, and substandard patient care. To our best knowledge, the performance of four AI-based chatbots—ChatGPT-4o, ChatGPT-4o Mini, Gemini, and Consensus—has never been compared and assessed on standardized clinical practice guidelines for NVO. Our strong study design with randomized presentation of questions, blinded expert grading, and independent review by three orthopedic surgeon colleagues strengthens the evidence. Among all models tested, ChatGPT-4o was the most consistently accurate and comprehensive, with Gemini a close second [21].

Previous studies comparing GPT-3.5 and GPT-4 demonstrated that these models were consistently capable of understanding clinical guidelines in clinical domains such as cervical radiculopathy, degenerative spondylolisthesis, lumbar spinal stenosis, and lumbar disc herniation [11,12]. Not trained for the purposes of specific clinical uses, these previous versions were also promising in terms of accuracy, provided proper oversight was exercised [13,14]. Our findings translate this pattern to the management of NVO, affirming that ChatGPT-4o and Gemini Advanced can provide guideline-applicable medical advice on a wide variety of spinal pathologies. Notably, ChatGPT-4o had the highest percentage of “Excellent” accuracy scores (54%), followed by Gemini at 51%. These findings align with earlier research pitting the models against the best practice guidelines of the American College of Surgeons and reporting a similarly high, but statistically undistinguishable, level of performance [15]. High internal consistency and a low rate of contradiction between ChatGPT-4o and Gemini indicate their logical coherence in complex clinical problem-solving tasks like antimicrobial selection, treatment planning, surgical considerations, and follow-up scheduling. In addition, ChatGPT-4o generated the longest responses, with an average of 428 words. This verbosity is in contrast to that generated using Chain-of-Thought (CoT) prompting in Med-PaLM 2, where longer responses were correlated with improved factual completeness [16]. Verbosity is not ideal in all cases, however. In clinical use cases where brevity and concision are critical, extremely long responses can make responses unusable. Balancing completeness and clinical usability, therefore, remains a critical design consideration for future-impending LLM deployment. ChatGPT-4o and Gemini both performed well across all three IDSA domains of diagnostics, therapy, and follow-up. ChatGPT-4o alone achieved a 100% “Excellent” accuracy metric in clinical therapy, which exemplifies its reliability in treatment-based scenarios. Gemini also matched the same strengths in therapy and follow-up recommendations. Despite high overall performance, we observed instances of potentially dangerous errors. For example, one model incorrectly recommended fluoroquinolone monotherapy for *Staphylococcus aureus* bacteremia-associated NVO, which contradicts IDSA guidelines advocating for prolonged IV therapy with agents like nafcillin or cefazolin. Another example involved the omission of follow-up imaging recommendations in cases with neurological deficits. These examples emphasize the need for careful clinical oversight when using LLM outputs. These findings were consistent with previous work showing good LLM performance in planning oncologic and surgical care [17]. Conversely, ChatGPT-4o Mini, despite having the same architecture as ChatGPT-4o, was less consistent. It produced primarily adequate output but lacked a lack of consistency and depth required in high-stakes decision-making. These results agree with other studies that the “Mini” forms of LLMs, while computationally economical, may fall short in reasoning-demanding applications [18]. Consensus Premium, designed for literature summarization, was both incomplete and inaccurate. While it could identify relevant sources, it did not use that evidence to make clinically relevant recommendations. This is indicative of its training in knowledge extraction rather than active clinical reasoning—a key distinction for the evaluation of AI systems that would be deployed in bedside decision-making [22].

### 4.1. Limitations

Certain limits must be remembered. Our work was founded on a static set of 13 standardized questions addressing only NVO. Performance will vary with various wordings, broader clinical domains, or more patient-tailored examples. Second, the research did not measure LLMs’ ability to handle real-time data or the most current literature—abilities increasingly important in rapidly evolving clinical spaces. Additionally, we did not assess the authenticity or correctness of references or citations provided by the LLMs. This introduces the risk of fictitious or biased citations, a known issue with some AI models. Future studies should systematically evaluate citation accuracy and related biases when LLMs are applied in clinical decision-making contexts.

Also, while grading was performed by senior spine surgeons, grading is inherently subjective. While blinding and consensus scoring minimized bias, future studies should employ automated benchmarking platforms to enhance objectivity. Another important limitation is the use of the 2015 IDSA guideline as a reference standard. Since LLMs were trained on data through 2021, inconsistencies may occur with stale reference material, which can result in confounding model accuracy assessment [23]. Lastly, our study utilized the 2015 IDSA guidelines, which, while still the most current for NVO, may not reflect recent advancements in diagnostics and therapeutics. This introduces a potential timeliness limitation. As guidelines are updated and LLM training data evolves, future research should evaluate model performance in handling new guideline iterations and their incorporation into LLM knowledge bases.

### 4.2. Practical Uses and Potential Future Paths

The implications of this study highlight the promise of highly performing LLMs, such as ChatGPT-4o and Gemini, to support a wide variety of clinical tasks. These involve interpretation of directives, triage support, clinical documentation, and care planning. In their optimal implementation, these tools can potentially reduce clinician workload, enhance the quality of decision-making, and facilitate care delivery [24,25,26]. For example, LLMs that are trained on specialty-specific information can generate clinical reports automatically that are tailored to spinal infections, e.g., critical diagnostic findings, treatment protocols, and postoperative plans. They can enhance multidisciplinary communication among infectious disease specialists, orthopedic surgeons, and rehabilitation teams. Moreover, incorporating LLMs into telemedicine workflows may extend access to subspecialist care for rural or underserved populations [27,28]. Prior instances in stroke triage and diabetic retinopathy screening demonstrate the potential of AI-supported diagnosis in primary care. Similarly, LLM-informed clinical decision-support systems could empower primary care providers to initiate evidence-based treatment, plan timely referrals, and follow-up with minimal delay [29,30]. While our study provides valuable insights into LLM performance in NVO, its findings may not generalize to other infectious diseases or broader clinical conditions. Extending similar evaluations to other spinal infections (e.g., epidural abscess, postoperative infections) and other infectious diseases could provide a more comprehensive understanding of LLM utility across clinical practice.

## 5. Conclusions

This paper describes a comprehensive evaluation of four LLMs—Consensus, Gemini, ChatGPT-4o Mini, and ChatGPT-4o—on interpreting and applying IDSA clinical guidelines for native vertebral osteomyelitis. Of the models tested, ChatGPT-4o recorded the highest overall accuracy, consistency, and completeness, followed by Gemini. Consensus and ChatGPT-4o Mini were more variable and less suitable for tasks requiring extensive clinical thought. While LLMs cannot replace human expertise, their integration into clinical workflows could enhance the delivery of timely, evidence-based care. Subsequent research must aim to conduct broader clinical evaluations, integrate data in real time, and analyze effects on medical practice and education. Once properly designed and validated, LLMs can be valuable assets for formulating medical practice and optimizing patient outcomes.

## Figures and Tables

**Table 1:** Average word count of LLM-generated responses to NVO-related clinical questions.

LLM		Response Length (Words)	
	Mean ± SD	Minimum	Maximum
**Consensus**	213.2 ± 68.8	43.0	305.0
**Gemini**	358.2 ± 60.5	273.0	475.0
**ChatGPT 4o Mini**	392.2 ± 97.35	179.0	543.0
**ChatGPT 4o**	428.0 ± 45.4	98.0	768.0

**Table 2:** Average character count of LLM-generated responses to NVO-related clinical questions.

LLM		Response Length (Characters)	
	Mean ± SD	Minimum	Maximum
**Consensus**	1313.0 ± 427.2	258.0	1929.0
**Gemini**	2223.0 ± 106.7	1616.0	2971.0
**ChatGPT 4o Mini**	2551.0 ± 175.6	1121.0	3249.0
**ChatGPT 4o**	2705.0 ± 295.3	624.0	5119.0

**Table 3:** Domain-specific accuracy score of LLM-chatbots to NVO-related questions.

Domain	No. of Questions	Model	Moderate	Good	Excellent
**Clinical Diagnostics**	21	Gemini	1 (5%)	13 (62%)	7 (33%)
		Consensus	4 (19%)	13 (62%)	4 (19%)
		ChatGPT 4o Mini	2 (9%)	10 (48%)	9 (43%)
		ChatGPT 4o	4 (19%)	9 (43%)	8 (38%)
**Clinical Therapy**	9	Gemini	0 (0%)	2 (22%)	7 (78%)
		Consensus	1 (11%)	5 (56%)	3 (33%)
		ChatGPT 4o Mini	0 (0%)	6 (66%)	3 (34%)
		ChatGPT 4o	0 (0%)	0 (0%)	9 (100%)
**Clinical Follow-up**	9	Gemini	0 (0%)	3 (33%)	6 (67%)
		Consensus	2 (22%)	6 (67%)	1 (11%)
		ChatGPT 4o Mini	0 (0%)	7 (78%)	2 (22%)
		ChatGPT 4o	1 (11%)	4 (44%)	4 (44%)

**Table 4:** Response comprehensiveness from LLM-chatbots to IDSA-related questions.

LLM		Response Comprehensiveness	
	n	Mean ± SD	Median
**Consensus**	13	2.87 ± 0.66	3.00
**Gemini**	13	3.82 ± 0.68	4.00
**ChatGPT 4o Mini**	13	3.15 ± 0.57	3.00
**ChatGPT 4o**	13	3.95 ± 0.79	4.00

**Table 5:** Inter-reviewer agreement statistics (Fleiss’ Kappa).

Evaluation Metric	Fleiss’ Kappa (κ)	Interpretation
Accuracy Scoring	0.61	Substantial Agreement
Comprehensiveness Scoring	0.57	Moderate Agreement

## Data Availability

Data are available from the corresponding author on reasonable request.

## Supplementary Materials

The following supporting information can be downloaded at https://www.mdpi.com/article/10.3390/jcm14144996/s1, Table S1: List of Clinical Questions Used in Model Evaluation.

## Abbreviations

The following abbreviations are used in this manuscript:

AI	Artificial Intelligence
ANOVA	Analysis of Variance
CoT	Chain of Thought
EMR	Electronic Medical Record
IDSA	Infectious Diseases Society of America
IRB	Institutional Review Board
LLM	Large Language Model
NASS	North American Spine Society
NVO	Native Vertebral Osteomyelitis
SD	Standard Deviation
