# Supplementary material for: Assessing LLMs on IDSA Practice Guidelines for the Diagnosis and Treatment of Native Vertebral Osteomyelitis: A Comparison Study
Source: J Clin Med. 2025 Jul 15;14(14):4996. doi: 10.3390/jcm14144996 (PMC12295083; doi:10.3390/jcm14144996)
Supplement: Supplementary file 1 [file jcm-14-04996-s001.zip › jcm-3736759-supplementary.pdf]

Supplementary material

(Supplementary Table S1): List of Clinical Questions Used in Model Evaluation

No.	Clinical Question
1	What are the typical clinical symptoms of native vertebral osteomyelitis (NVO)?
2	Which laboratory tests are essential for diagnosing NVO?
3	What is the most appropriate imaging modality for diagnosing NVO?
4	How should microbiological sampling be performed in suspected NVO cases?
5	When should a percutaneous biopsy be considered in NVO diagnosis?
6	What are the common causative pathogens of NVO?
7	What are the first-line antibiotic therapies recommended for NVO?
8	How long should antibiotic therapy be continued for NVO?
9	When is surgical intervention indicated for NVO?
10	How should treatment be monitored during NVO therapy?
11	What are the recommended criteria for treatment success in NVO?
12	How should recurrent or relapsing NVO be managed?
13	What are the recommended follow-up imaging protocols after NVO treatment completion?
